# Cellular and Molecular Targets of Waterbuck Repellent Blend Odors in Antennae of *Glossina fuscipes fuscipes* Newstead, 1910

**DOI:** 10.3389/fncel.2020.00137

**Published:** 2020-06-03

**Authors:** Souleymane Diallo, Mohd Shahbaaz, Baldwyn Torto, Alan Christoffels, Daniel Masiga, Merid N. Getahun

**Affiliations:** ^1^International Centre of Insect Physiology and Ecology, Nairobi, Kenya; ^2^South African Medical Research Council Bioinformatics Unit, South African National Bioinformatics Institute, University of the Western Cape, Cape Town, South Africa; ^3^Department of Zoology and Entomology, University of Pretoria, Hatfield, South Africa

**Keywords:** behavior, molecular docking, molecular dynamics, olfaction, physiology, repellents, tsetse

## Abstract

Insects that transmit many of the world’s deadliest animal diseases, for instance trypanosomosis, find their suitable hosts and avoid non-preferred hosts mostly through olfactory cues. The waterbuck repellent blend (WRB) comprising geranylacetone, guaiacol, pentanoic acid, and δ-octalactone derived from waterbuck skin odor is a repellent to some savannah-adapted tsetse flies and reduces trap catches of riverine species. However, the cellular and molecular mechanisms associated with detection and coding of the repellent odors remain to be elucidated. Here, we demonstrated that WRB inhibited blood feeding in both *Glossina pallidipes* Austen, 1903 and *Glossina fuscipes fuscipes* Newstead, 1910. Using the DREAM (*D*eorphanization of *R*eceptors based on *E*xpression *A*lterations in odorant receptor *m*RNA levels) technique, combined with ortholog comparison and molecular docking, we predicted the putative odorant receptors (ORs) for the WRB in *G*. *f*. *fuscipes*, a non-model insect. We show that exposure of *G*. *f*. *fuscipes in vivo* to WRB odorant resulted in up- and downregulation of mRNA transcript of several ORs. The WRB component with strong feeding inhibition altered mRNA transcript differently as compared to an attractant odor, showing these two odors of opposing valence already segregate at the cellular and molecular levels. Furthermore, molecular dynamics simulations demonstrated that the predicted ligand–OR binding pockets consisted mostly of hydrophobic residues with a few hydrogen bonds but a stable interaction. Finally, our electrophysiological response showed the olfactory sensory neurons of *G*. *f*. *fuscipes* tuned to the tsetse repellent components in different sensitivity and selectivity.

## Introduction

Blood-feeding insects such as tsetse flies have a differential feeding preference to some animals over others regardless of their abundance (Weitz, 1963). Such behavior is a response to odors and can lead to the identification of attractants and repellents for vector control. The spatial repellent for some tsetse fly species is a blend of semiochemicals formulated to prevent tsetse flies from encountering a livestock host from distance, identified from a non-host, waterbuck (*Kobus defasa*) (Gikonyo et al., 2002, 2003; Mwangi et al., 2008). Such formulations can reduce encounters between livestock and vectors, thereby eliminating or reducing the probability (risk) of pathogen transmission. The repellent formulation is a blend that consists of δ-octalactone, guaiacol, geranyl acetone, and pentanoic acid (Bett et al., 2015; Saini et al., 2017). Until recently, the main tools for tsetse control were odor-baited traps and targets (Kuzoe and Schofield, 2004). Additionally, recent use of the tsetse repellent blend (waterbuck repellent blend [WRB]) has reduced the transmission of animal trypanosomiasis in cattle by reducing contact between blood-seeking *Glossina pallidipes* and cattle hosts (Saini et al., 2017) and has been shown to reduce trap catches of *Glossina fuscipes fuscipes* (Mbewe et al., 2019). The cellular and molecular mechanisms of the spatial repellent odors are not well understood, yet such knowledge will enable us to improve the efficacy of the existing repellent blend or to identify novel repellents for control of various tsetse fly species of both medical and veterinary importance. Olfactory research on *Glossina* spp. is fragmented (Chahda et al., 2019; Soni et al., 2019). However, recent research on tsetse genomes has opened new opportunities to make functional characterization of *Glossina* odorant receptors (ORs) possible (Aksoy et al., 2014; [Bibr B53][Bibr B76]; Macharia et al., 2016; Attardo et al., 2019).

In *Drosophila*, since the first insect ORs were identified (de Bruyne et al., 1999; Vosshall et al., 1999), enormous progress has been made to functionally characterize almost the entire OR repertoire and elucidate the molecular basis of olfaction in this insect species (Hallem et al., 2004). The limited genetic tools available for non-model insects has limited functional characterization studies of ORs in economically important insects such as tsetse flies. A recently developed technique (Weid et al., 2015) that compares change in mRNA due to odor stimulation could be a useful tool to identify potential OR genes in non-model insects. In this technique, when living insects were exposed to a given odorant, the expression levels of mRNA transcript of ORs activated by the odor were altered; some were upregulated, with others downregulated (Weid et al., 2015; Koerte et al., 2018).

Bett et al. (2015) demonstrated that a five-component blend (WRB) was enough to repel tsetse flies significantly, and that each component contributed differently to the repellency. The main aim of the present study was to describe the cellular and molecular bases of WRB and component coding using *G. f*. *fuscipes*, an important vector of both human and African animal trypanosomiasis, based on activity-dependent changes in OR mRNA transcripts. Here, we show that exposure of *G*. *f*. *fuscipes* to the tsetse repellent blend changes the mRNA transcript of several ORs and that the blend components elicit a strong antifeedant behavior and physiological response in *G*. *f*. *fuscipes*.

## Materials and Methods

### Biological Material

The tsetse flies used in this study were obtained from the colony maintained at the insectary of the International Centre of Insect Physiology and Ecology (*icipe*). The flies were maintained at 24 ± 1°C and 75–80% relative humidity and were fed three times per week by membrane feeding with defibrinated bovine blood collected from a local slaughterhouse.

### Antifeeding Bioassay

This experiment was performed using non-teneral flies (9–10 days old). Before the antifeeding bioassay, the flies were starved for 3 days. *In vitro* feeding was done using a silicone membrane feeding system following standard mass rearing procedures (Feldmann, 1994; Food and Agriculture Organization of the United Nations/International Atomic Energy Agency [FAO/IAEA], 2006). To prepare the treated membrane, 100 μL of the diluted chemical at 10^–3^ (v/v) or the solvent was applied on 2116 cm^2^ of a silicone membrane. The four-component WRB comprised δ -octalactone, geranylacetone, guaiacol, and pentanoic acid roughly in a ratio of 3:1:2:3, respectively, as found naturally in the waterbuck odor (Gikonyo et al., 2002). The chemicals were first loaded into the feeding membrane and spread to the whole surface of the membrane using cotton wool. The feeding started 5 min later after the application of the chemical on the silicone membrane when the solvent had evaporated. For each treatment, flies of the same age were separated into two groups of 20 flies each in a 1:1 sex ratio. The first group was fed on the treated silicone membrane, while the second group (control group) was fed on membrane treated with solvent only. The feeding of the two groups was simultaneously done, and for each group, flies were individually fed and weighed before and after feeding. The feeding efficiency was calculated by the difference in weight of the individual fly before and after feeding. Using the feeding efficiency, the feeding index (FI) was calculated as (T−C)/(T + C), with T representing the amount of blood taken by the fly when a membrane is treated with given compound and C representing the amount of blood taken by the fly on an untreated membrane (solvent only). As previously done by Dweck et al. (2013) and Ebrahim et al. (2015), deviation of the FI from zero was tested with a Student’s *t*-test (*P* < 0.05). The distribution of the data was checked using the Shapiro test. Student’s *t*-test followed by Cohen’s *d* test were performed on independent samples corresponding to different treatments. Multiple testing was not performed in any sample; hence no *P*-value adjustment was required. The statistics were generated using R software, version 3.5.1 (R Core Team, 2018), www.R-project.org.

### Single-Sensillum Recording

Both male and female *G*. *f*. *fuscipes*, 5–7 days old and starved for 2 days, were used. The single-sensillum recording was performed as described previously (de Bruyne et al., 1999; Hallem et al., 2004; Getahun et al., 2012; Chahda et al., 2019; Soni et al., 2019). Only one recording was made from a single fly to avoid response adaptation from multiple stimulations. Briefly, the flies were mounted in a cut pipette tip (blue) with the head protruding and a small amount of wax placed at the back of the tip to prevent retraction of the fly. The pipette was then fixed onto a microscope slide with wax, and the antennae fixed on a coverslip with a sharpened glass electrode. A sharpened tungsten electrode was placed in the eye for grounding, and a second recording electrode was brought in to contact with the base of the sensillum using a PM 10 Piezo manipulator. The electrodes were sharpened using saturated potassium nitrite (KNO_2_) solution. The sensilla were observed with an Olympus BX-51WI microscope (Olympus Corporation, Tokyo, Japan) at 1000× magnification. Odorants were diluted in dichloromethane (DCM) at 10^–3^ (v/v) except for pentanoic acid, which was diluted in distilled water at 10^–3^ (v/v). We selected the concentration within the linear portion of the dose–response curve from recent study on tsetse fly study (Chahda et al., 2019). Then 10 μL of the diluted odorant were pipetted on 1-cm-diameter filter paper disk placed in glass Pasteur pipettes. Flies were stimulated by placing the tip of a cartridge into a glass tube that delivered a stream of humidified air (0.5 L/min) to the fly’s antenna (Getahun et al., 2012). The odors were delivered by puffing them using the Syntech stimulus delivery system. The odor stimulus was administered as a 0.5-s pulse of charcoal-filtered air (5.9 mL/s) by placing the tip of the glass Pasteur pipette through a hole in a tube carrying a purified air stream. The signal was amplified (Syntech universal AC/DC 10X probe^1^) and digitally converted (SyntechIDAC-4). The responses (spikes/s) were analyzed by counting the number of spikes, 1 s during the 0.5-s stimulation minus 1 s before stimulation offline using AutoSpike software (Olsson and Hansson, 2013). We used AutoSpike software for identification of responding neurons (Syntech^2^). The activity of colocated olfactory sensory neurons (OSNs) in single sensilla was differentiated based on differences in their spike amplitude. As spike amplitudes sometimes change during extensive firing when stimulated with odorant, we had to complement the static template used by the software for spike sorting with manual sorting, whereby attention was also paid to the shape of the spikes. Responses of individual OSNs were calculated as the increase (or decrease) in impulse rate (spikes per second) relative to the prestimulus rate. Each sensillum was tested with all odorants. We used AutoSpike v3.9 signal acquisition software (Syntech Ockenfels, Germany).

### Chemicals

The synthetic chemicals geranylacetone, δ-octalactone, guaiacol, pentanoic acid, and 1-octen-3-ol were purchased from Sigma-Aldrich at the highest available purity. Geranylacetone, δ-octalactone, guaiacol, and 1-octen-3-ol were diluted in absolute ethanol (99.8%) (Conde, 2014; Karlsson and Friedman, 2017) and pentanoic acid in distilled water.

### Odorant Exposure and RNA Extraction

The flies were exposed to different odorants of 10^–3^ (v/v), the concentration within the linear portion of the dose–response curve, to minimize false-positive and false-negative results for 5 h (Weid et al., 2015; Koerte et al., 2018) in a Plexiglas cubic cage (13.5 cm × 13.5 cm × 20 cm). To avoid any mating, males and females were exposed in a separate cage under mass-rearing conditions and 25 flies were placed per cage for the odorant exposure. After exposure, flies were chilled at −80°C for 5 min and their antennae were removed on ice. The main reason why we targeted antenna is because the WRB is a spatial repellent that is detected predominantly by the antennae. Antennae were removed from 150 flies (male–female ratio 1:1) representing three biological replicates. Dissected antennae of male and female were mixed and collected in 2.0-mL microcentrifuge tubes. The microcentrifuges were stored in liquid nitrogen during the antennae dissection to preserve the integrity of RNA transcripts. After dissection, samples were homogenized with a bead mill using Tissue Lyser LT (Qiagen) for 10 min at 50 Hz. The samples were centrifuged at 13,000 *g* for 5 min, and 350 μL of the homogenate was used for total RNA isolation. Total RNA was isolated using TRIzol reagent (Invitrogen, Thermo Fisher Scientific), following the manufacturer’s instructions. RNA concentration and purity were evaluated using a spectrophotometer (GeneQuant Pro RNA/DNA calculator, Amersham Biosciences, Cambridge, United Kingdom) measuring absorbance at A260 and A280 nm. Before converting to cDNA, RNA was temporarily stored at −80°C in nuclease-free water.

### Quantitative Real-Time Reverse Transcription Polymerase Chain Reaction Assay and Data Analysis

The total RNA was reverse transcribed from 500 ng in a 20-μL reaction mixture using the High Capacity cDNA Reverse Transcription kit (Applied Biosystems, Foster City, CA, United States) according to the manufacturer’s instructions. The cDNA was amplified in 12.5 μL of 1 × SYBR Green Master Mix (Applied Biosystems) according to the manufacturer’s instruction. The primers (Supplementary Table S1) sets were designed with Primer3 software and optimized with gradient polymerase chain reaction (PCR) using Kyratec Thermal cycler). The quantitative PCR (qPCR) experiment was performed with QuantStudio 3 using the comparative ΔΔC_T_ method as previously described (Bustin and Nolan, 2004). A previous study (Koerte et al., 2018) used *Orco* and *CAM* as reference genes and found that their mRNA transcripts levels could be altered by the exposure to chemicals. It was then suggested that the choice of *Orco* as the reference gene might be one of the factors that could affect the efficiency of the DREAM technique. Briefly, DREAM refers to *D*eorphanization of *R*eceptors based on *E*xpression *A*lterations of OR *m*RNA levels), which allows us to identify the chemosensory receptors interacting with an odorant in a high-throughput manner instead of a deorphanization of single ligand–receptor pairs at a time. The method is based on the comparison of the mRNA transcript levels of ORs between treated (exposed insects) and control (unexposed) insects using reverse transcription (RT)-qPCR. If an odor interacts with receptors its mRNA is altered, either up- or downregulated, whereas mRNA remains the same for ORs that do not interact with the odors (Weid et al., 2015; Koerte et al., 2018). Hence, in this study, we used *b-actin* as the reference gene for our ΔΔC_T_ calculation.

### Ortholog Comparison and *in silico* Prediction of Ligand–Receptor Interactions

Orthologs of ORs were identified using Vectorbase^3^ and Flybase^4^. The receptor response profiles in *Drosophila melanogaster* were identified in the Database of Odorant Responses (DoOR) (Silbering et al., 2008; Ebrahim et al., 2015).

Homology modeling of the studied proteins was performed using the fold recognition algorithm present in Phyre2 server (Kelley et al., 2015). The “Intensive mode,” which combines the *ab initio* techniques, was used to perform complete modeling of the entire proteins. The olfactory coreceptor (*Orco*) structure (PDB ID: 6C70) from *Apocrypta bakeri* (Butterwick et al., 2018) was used as a template for structure predictions. The template structure was obtained at its high resolution (3.5 Å) from Protein Data Bank (Berman et al., 2000). The quality of our predicted models was evaluated using SAVES v5.0^5^ tools (Supplementary Table S2).

Predicted 3-D models were optimized and molecular docking was performed using ICM-Pro software (Ruben Abagyan, 1994) version 3.8.7 (MolSoft LCC, San Diego, CA, United States^6^ The binding pockets were identified using ICM Pocket Finder before the molecular docking. The binding pocket was chosen within the 2-extracellular and 3-extracellular loop (Lua et al., 2016; Batra et al., 2019). Membrane topologies were analyzed using psipred-MEMSATSVM^7^.

### Molecular Dynamics Simulations

For each WRB component, the best scoring complex was selected and subjected to molecular dynamics (MD) simulations using GROningen MAchine for Chemical Simulations (GROMACS) 5.1.2 (Pronk et al., 2013). However, for δ- octalactone, we included one more complex with a different scoring given that we used its analogs as reference in Drosophila receptors. Primarily, the GROningen MOlecular Simulation (GROMOS) 96 53a6 force field (Oostenbrink et al., 2004) was used to generate the topologies of the protein structures in the docking based generated different complexes. Moreover, the topologies of the studied ligand compounds were generated using the PRODRG server (Schüttelkopf and Van Aalten, 2004). But the PRODRG does not contain the server functionality of generating the partial charges of the studied ligands; therefore, the Density Functional Theory (DFT) method implemented in GAUSSIAN that utilized the B3LYP 6-31G (d,p) basis set and the CHarges from ELectrostatic Potentials using a Grid (CHELPG) program (Frisch et al., 2009) was used for correction. After successful topology generation of the docked complexes, the complexes were solvated using the SPC/E water model (Zielkiewicz, 2005) and then neutralized by adding a suitable number of sodium (Na) and chlorine (Cl). Consequently, the systems were subjected to an energy minimization step using the combined steepest descent as well as conjugate gradient algorithms, with a convergence criterion of 0.005 kcal/mol. Before the equilibration step the position restraints were applied to the structure of the ligands in the minimized system ligands (Idrees et al., 2018; Shahbaaz et al., 2018, 2019).

The equilibration step was carried out into the combined stages of NVT (constant volume) and NPT (constant pressure) ensemble conditions, each at a 100 ps time scale. The temperature of 300 K was maintained for the system using the Berendsen weak coupling method, and pressure of 1 bar was maintained utilizing Parrinello–Rahman barostat in the equilibration stage. The LINear Constraint Solver (LINCS) algorithm was used for the generation of final conformational production stage for the 100 ns timescale, and trajectories were generated, which were analyzed in order to understand the behavior of each complex in the explicit water environment. The changes in the H-bonds, as well as the root mean square deviations (RMSDs) and radius of gyration (Rg) of the complex systems were analyzed (Idrees et al., 2018; Shahbaaz et al., 2019). Furthermore, the molecular mechanics Poisson–Boltzmann surface area (MM-PBSA) protocols implemented in the g_mmpbsa package (Kumari et al., 2014) was used for the calculation of free energy of binding protein and the ligand molecules.

## Results

### Repellent Odorants Reduce Tsetse Fly Blood Feeding

**FIGURE 1:**
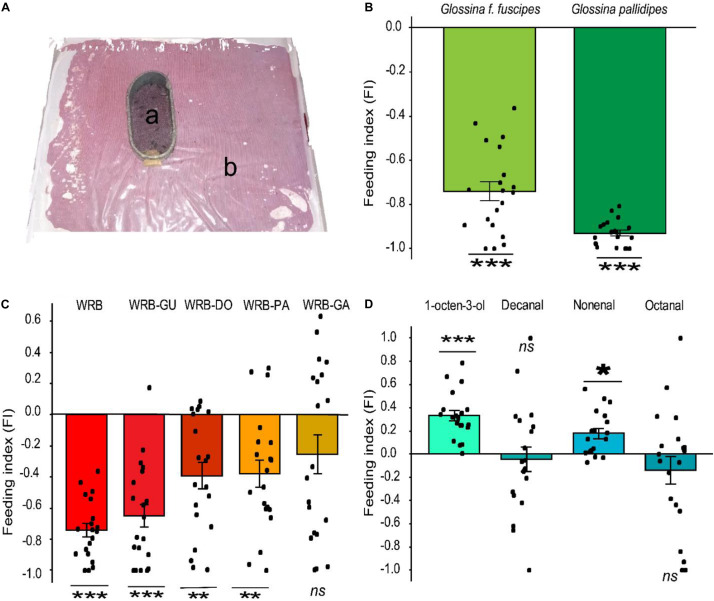
Antifeeding effect of WRB. **(A)** Antifeeding bioassay setup: *a* represents the feeding cage, single fly/cage (original photo); *b* indicates the feeding tray containing sterile blood covered by a silicone membrane. **(B)** Feeding index (FI) of *G*. *f*. *fuscipes* and *G*. *pallidipes* fed on membrane treated with WRB. WRB, waterbuck repellent blend. Deviation of the feeding index from zero was tested with a Student’s *t*-test (*P* < 0.05). **(C)** Feeding index (FI) of *G*. *f*. *fuscipes* fed on membrane treated with WRB and showing the contribution of each compound to the antifeeding effect. WRB-DO, WRB minus δ-octalactone; WRB-GA, WRB minus geranyl acetone; WRB-GU, WRB minus guaiacol; WRB-PA, WRB minus pentanoic acid. **(D)** Feeding index (FI) of *G*. *f*. *fuscipes* fed on membrane treated with positive controls (an attractant). Deviation of the feeding index from zero was tested with a Student’s *t*-test (*P* < 0.05). Level of significance: ***P < 0.0001; **P < 0.001; *P < 0.05, *d* represents the effect of size (Cohen’s *d*) and ns means not significant. Error bars represent standard error, *n* = 20 for each test. The graphs and the statistics were generated using R software (R Core Team, 2018), (version 3.5.1), www.R-project.org.

Since WRB has a strong spatial repellent effect on *G*. *pallidipes* and reduced the contact between the vector and the host (Bett et al., 2015; Saini et al., 2017), we tested if it also influenced the blood feeding behavior of this tsetse fly species and the related species *G*. *f*. *fuscipes* (Figure 1A). We found that the feeding behavior was significantly inhibited in *G*. *pallidipes* (*t*-test, *P* = 2.2e-16, *d* = 15.73, *n* = 20) relative to the control. Likewise, the feeding behavior of *G*. *f*. *fuscipes* was also inhibited (*t*-test, *P* = 5.08e-13, *d* = 3.83, *n* = 20) (Figure 1B). The FI of the flies fed on the treated membrane was −0.93 and −0.74 in *G*. *pallidipes* and *G*. *f*. *fuscipes*, respectively. We then tested the contribution of each component of the WRB in this antifeeding effect in subtractive assays. Removal of guaiacol from the blend did not affect the feeding inhibition in *G*. *f*. *fuscipes* flies (FI = −0.65, *t*-test, *P* = 3.356e-08, *d* = 1.98). However, removal of pentanoic acid or δ-octalactone significantly reduced the antifeeding effect in this tsetse fly species compared to the antifeedant activity elicited by the full blend. The FI of flies fed on membrane treated with WRB minus pentanoic acid was −0.38 (*t*-test, *P* = 0.002755, *d* = 0.99) and −0.39 (*t*-test, *P* = 0 0.001772, *d* = 1.03) for WRB minus δ-octalactone (Figure 1C). On the other hand, removal of geranylacetone from the WRB significantly reduced the antifeedant effect of the blend, that is, the flies fed more, demonstrating that GA was a key component for the feeding deterrence effect of the WRB (FI = −0.26, *t*-test, *P* = 0.05536, *d* = 0.45) (Figur 1C).

Next, we assessed whether the blood feeding inhibition could be due to the presence of novel odors on the membrane. To do this, we tested the known tsetse fly attractant 1-octen-3-ol and preferred host (Buffalo/ox) volatiles nonanal, decanal, and octanal (Gikonyo et al., 2002) as a positive control in identical assays. We found that decanal and octanal had no effect on the feeding efficiency, i.e., no inhibition or enhancement. However, nonanal and 1-octen-3-ol enhanced the feeding efficiency in *G*. *f*. *fuscipes* compared to the control (FI = 0.33; *t*-test, *P* = 0.004312, *d* = 1.36) (Figure 1D). These results confirm that the feeding inhibition in Figures 1B,C was not due to the presence of novel odors on the membrane, but due to the presence of specific odors, in our case the WRB blend.

### Exposure to Tsetse Repellent Odorants Induced Change in Receptors of mRNA Transcript Level

**FIGURE 2:**
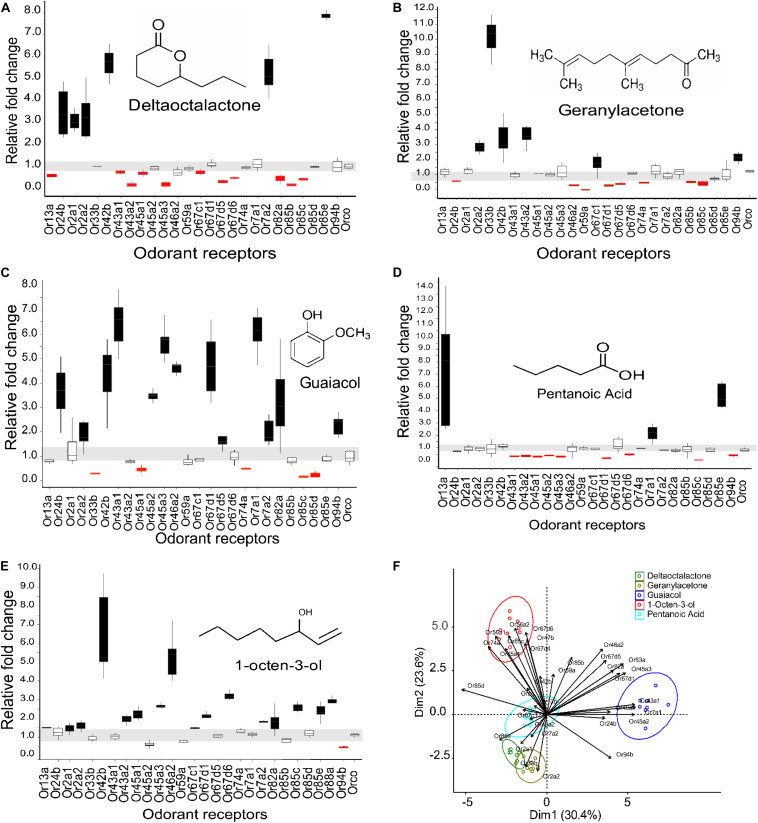
Expression pattern of *G*. *f*. *fuscipes* odorant receptors after exposure to WRB components and 1-octen-3-ol. The horizontal gray zone corresponds to OR mRNA transcript values that were not affected. **(A)** Expression pattern of ORs to δ-octalactone. **(B)** Expression pattern of ORs to geranylacetone. **(C)** Expression pattern of ORs to guaiacol. **(D)** Expression patterns of ORs to pentanoic acid. **(E)** Expression pattern of ORs to 1-octen-3-ol. **(F)** PCA plot showing the clustering pattern of the five tested ligands based on the fold change of the mRNA of ORs **(A–E)**. The PCA explained 53.6% of the total variation. ORs that do not fall into one of the cluster circles **(F)** show that the mRNA transcript level was not affected by the odorant exposure. The graphs and the statistics were generated using R software21 (version 3.5.1), www.R-project.org. (PCA) was performed using two R packages called “FactoMineR” and “Factoextra” (Kassambara, 2017).

**FIGURE 3:**
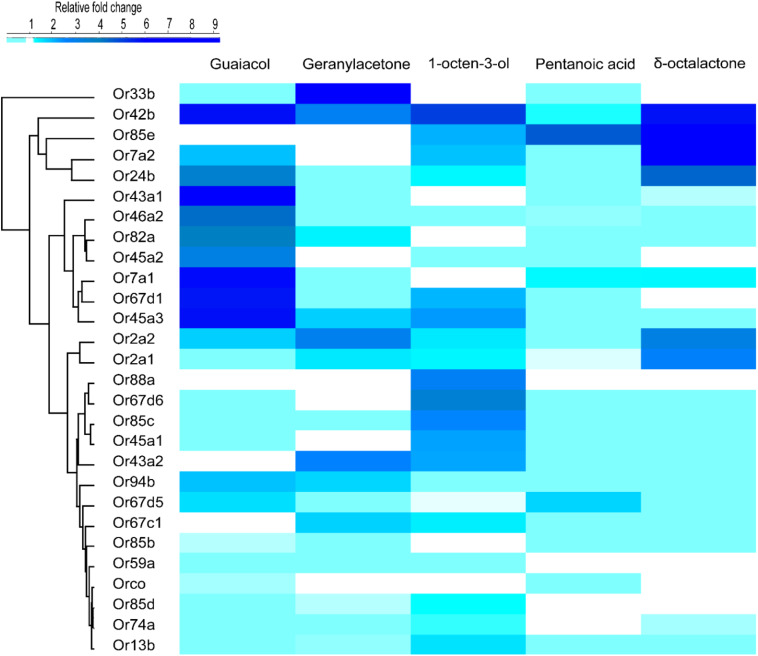
Heat map showing the differential expression of *G*. *f*. *fuscipes* odorant receptors across the five odorants, generated using R software (R Core Team, 2018) (version 3.5.1) www.R-project.org, edited using adobe illustrator CS5.1.

We used activity dependent change of mRNA transcript level (Weid et al., 2015; Koerte et al., 2018) in 27 ORs in *G*. *f*. *fuscipes* to identify potential receptors of WRB and 1-octen-3-ol. These 27 ORs were selected because of their PCR efficiency during optimization of primers. We used the WRB components δ -octalactone, geranylacetone, guaiacol, and pentanoic acid, mixed at the ratio of 3:1:2:3, respectively according to their abundance in waterbuck odor (Gikonyo et al., 2002, 2003; Mwangi et al., 2008; Bett et al., 2015) and the attractant 1-octen-3-ol (Hall et al., 1984; Vale and Hall, 1985), which has a different odor valence in this experiment. We found that the mRNA transcripts of ORs were differentially affected by the various odorant exposure after 5 h. The exposure of flies to δ-octalactone induced downregulation of six OR mRNA; however, nine OR mRNA transcripts were upregulated (Figure 2A). The exposure to geranyl acetone altered the mRNA transcripts levels of 20 ORs in total, whereby 9 were upregulated with 11 downregulated (Figure 2B). Exposure to guaiacol affected the transcript levels of 22 ORs; they were upregulated in 15 ORs downregulated in 7 ORs (Figure 2C). However, the numbers of ORs that were up- and downregulated were not significantly different (*P* > 0.05). Exposure to pentanoic acid affected 14 ORs mRNA transcripts; 4 were upregulated, whereas 10 were downregulated (Figure 2D). In contrast, the attractant chemical 1-octen-3-ol significantly upregulated mRNA transcripts of 21 ORs, but downregulated mRNA of only one OR transcript (GffOr94b) (Figure 2E), (χ^2^, 18.8, df = 1, *P* < 0.0001). The gene expression patterns are well represented in the heatmap (Figure 3). The positive control gene, i.e., the coreceptor *Orco* mRNA expression, was not affected by the odor exposure in all exposed flies (Figures 2A–E).

To correlate feeding inhibition and OR mRNA gene expression alteration, we performed a principal component analysis (PCA). The PCA was based on the log fold change in OR mRNA transcript expression. The PCA analysis separated the three components (geranylacetone, δ-octalactone, and pentanoic acid) that significantly deterred fly feeding from the blend component (guaiacol) that had no effect on feeding. The OR mRNA transcript alteration pattern of 1-octen-3-ol, a known attractant and feeding stimulant according to this study, was clearly discriminated by PCA from the other odorants that inhibited blood feeding (Figure 2F). To correlate the antifeedant effect with mRNA transcript alteration, WRB components with strong feeding inhibition affected an almost equal number of OR mRNA transcripts by up- and downregulation. However, the two compounds, 1-octen-3-ol and guaiacol, that elicited no detectable feeding inhibition induced overexpression of OR mRNA transcripts in several ORs as compared to those downregulated, but that by guaiacol is not significantly different.

### Comparison of the Response Profile of *G*. *f*. *fuscipes* Receptors to Their Orthologous Receptors in *Drosophila melanogaster*

We aimed to compare the ligand–receptor pairing in our study to their deorphanized orthologs in *D. melanogaster*, which has functionally well characterized ORs for comparison with *G*. *f*. *fuscipes* OR orthologs genes (Supplementary Table S3). For *D*. *melanogaster*, there is an online platform (DoOR) that provides an extensive database for known ligand–OR pairs.

In our activity-dependent change in mRNA expression, exposure to pentanoic acid affected many OR mRNA transcripts, in which most were downregulated and a few upregulated. Similarly, in *D*. *melanogaster* pentanoic acid activates several ORs (DoOR). Comparing our data to pentanoic acid (Kreher et al., 2005; Hallem and Carlson, 2006; Silbering et al., 2008; Galizia and Rössler, 2010), we found substantial similarity between 9 ORs whose mRNA expression were altered: GffOr7a1, GffOr7a2, GffOr13a, GffOr43a1, GffOr43a2, GffOr43a3, GffOr45a1, GffOr45a2, and GffOr63a in *G*. *f*. *fuscipes* (Figures 2D, 3) and their orthologs in *D*. *melanogaster* (DoOR). In the DoOR database, only DmOr19b has been reported as a receptor of geranylacetone in *D*. *melanogaster*. Its ortholog in *G*. *f*. *fuscipes*, which is GffOr2a2, also elicited a change in mRNA expression level to geranylacetone exposure. Additionally, other receptors of *G*. *f*. *fuscipes* were affected by the geranylacetone exposure (Figures 2B, 3). The orthologs of the remaining *G*. *f*. *fuscipes* ORs that responded to geranylacetone in our study were not reported as receptors of geranylacetone in the DoOR database. Guaiacol has four receptors in *D*. *melanogaster* (DmOr7a, DmOr19b, DmOr22a, and DmOr71a), according to previous studies (Stensmyr et al., 2003; Dweck et al., 2015) GffOr7a1 and GffOr7a2 orthologs of DmOr7a, GffOr42b ortholog of DmOr22a, and GffOr2a1 ortholog of DmOr19b were all upregulated after exposure to guaiacol in our study. The following ORs of *D*. *melanogaster*, DmOr47b, DmOr33b, DmOr35a, and DmOr85b, are orthologs of GffOr47b, GffOr33b, GffOr74a, and GffOr85c, respectively did not respond to guaiacol but elicited a response to the related the compound, 4-ethylguaiacol.

The ORs for δ-octalactone are not reported yet in DoOR; however, the receptors for some of its analogs have been documented in the DoOR database. Comparing the G. *f*. *fuscipes* ORs affected by δ-octalactone exposure to the response profile of some of the orthologs in *D*. *melanogaster*, we found similarities between *D*. *melanogaster* Or35a, Or19a, and Or22a with GffOr85b, GffOr45a3, GffOr24b, and GffOr7a2, as potential receptors of δ-octalactone in *G*. *f*. *fuscipes*. The attractant compound 1-octen-3-ol affected several OR mRNA transcripts in most of them by upregulation (Figures 2E, 3). Similarly, in *D*. *melanogaster*, 1-octen-3 -ol activated many ORs. The change in mRNA transcripts of GffOr13a, GffOr42b, and GffOr88a match with the following orthologs ORs in *D*. *melanogaster*, DmOr43a2, DmOr43a1, and DmOr59a, which are 1-octen-3-ol receptors, showing GffOr13a, GffOr42b, and GffOr88a are potential 1-octen-3-ol receptors in *G*. *f*. *fuscipes*.

### *In silico* Prediction of Ligand–Odorant Receptor Interaction

We further compared the response profile of *G*. *f*. *fuscipes* ORs with *D*. *melanogaster* ORs using molecular docking to predict the potential ORs and ligand interactions. Briefly, molecular docking is a computation that predicts the preferred interactions between two molecules, usually a protein and its ligand molecule(s). The docking simulation predicts the optimized binding parameters of ligand–receptor complexes. When two molecules interact, the resulting complex will be stable when it has lower free binding energy (< < 0). Conversely, an unstable protein complex will have a higher free binding energy (> > 0). Hence, the more negative the number generated for the free energy (docking score), the more efficient the interaction between the two molecules and by extension the more stable the complex. In *G*. *f*. *fuscipes*, all the receptors whose OR expressions were upregulated or downregulated after exposure to the WRB components were docked within extracellular loop-2 and -3. Before ligand–odorant interaction studies, we checked the topology of the ORs using psipred-MEMSATSVM^8^.

As *D*. *melanogaster* ORs are well deorphanized, we first identified the best receptors for our ligand in DoOR database. We compared the binding affinity score of *G*. *f*. *fuscipes* to *D*. *melanogaster* receptor binding scores. The binding affinity scores were considered as reference. Since no receptors have been reported for δ-octalactone in the DoOR database, we chose its analog compounds γ-octalactone and hexa-octalactone receptors, DmOr35a, DmOr19b, and DmOr22a in *D*. *melanogaster*. The binding affinities of these receptors with δ-octalactone were −22.14, −16.59, and −12.59 kcal/mol, respectively. In *G*. *f*. *fuscipes*, five receptors showed similar binding affinity (Table 1). DmOr19b is known as the receptor of geranyl acetone, the only reported receptor. Its binding affinity with its ligand is −14.15 kcal/mol. In *G*. *f*. *fuscipes*, GffOr2a2, GffOr59a, and GffOr33b interacted with geranylacetone (Table 1). We found almost equal binding affinity (env. −15.5 kcal/mol), when we docked guaiacol to three of its receptors in *D*. *melanogaster*. In *G*. *f*. *fuscipes*, guaiacol showed high binding affinity of −25.84, −16.43, and −15.65 kcal/mol with GffOr46a2, GffOr67d1, and GffOr45a3, respectively. *In D*. *melanogaster*, we selected DmOr7a, DmOr22a, and DmOr71a as reference receptors for pentanoic acid. The docking of pentanoic acid to the selected receptors showed a binding energy between −15.75 and −25.84 kcal/mol. In *G*. *f*. *fuscipes*, we found that four receptors have similar or higher binding efficiency (Table 1). 1-Octen-3-ol is known to be detected by several receptors. Based on the binding score of three receptors of *D*. *melanogaster*, we identified GffOr13a, GffOr42b, and GffOr88a as potential receptors of 1-octen-3-ol in *G*. *f*. *fuscipes*.

**TABLE 1:** Docking scores of the potential receptors for WRB components and 1-octen-3-ol identified in *G*. *f*. *fuscipes* through ligand–receptor interactions.

Ligands	*Drosophila melanogaster*		*Glossina fuscipes fuscipes*	
		
	Docking score (kcal/mol)	ORs	Docking score (kcal/mol)	ORs
δ-Octalactone	−22.14	Or35a	−21.51	Or85b
	−16.59	Or19a	−17.24	Or45a3
	−12.59	Or22a	−16.19	Or24b
			−14.33	Or7a2
Geranyl acetone	−14.15	Or19b	−16.38	Or2a2
			−15.21	Or59a
			−14.11	Or33b
Guaiacol	−15.33	Or7a	−25.84	Or46a2
	−15.52	Or22a	−16.43	Or67d1
	−15.52	Or71a	−15.65	Or45a3
Pentanoic acid	−16.53	Or7a	−25.84	Or45a2
	−16.20	Or45a	−21.21	Or67d6
	−15.75	Or67a	−21.51	Or43a1
			−15.82	Or67d1
1-Octen-3-ol	−17.51	Or43a2	−19.94	Or13a
	−11.31	Or43a1	−18.77	Or42b
	−10.63	Or59a	−17.62	Or88a

### Molecular Dynamic Simulations

#### Hydrogen Bonding Pattern of Docked ORx–Ligand Complexes

**FIGURE 4:**
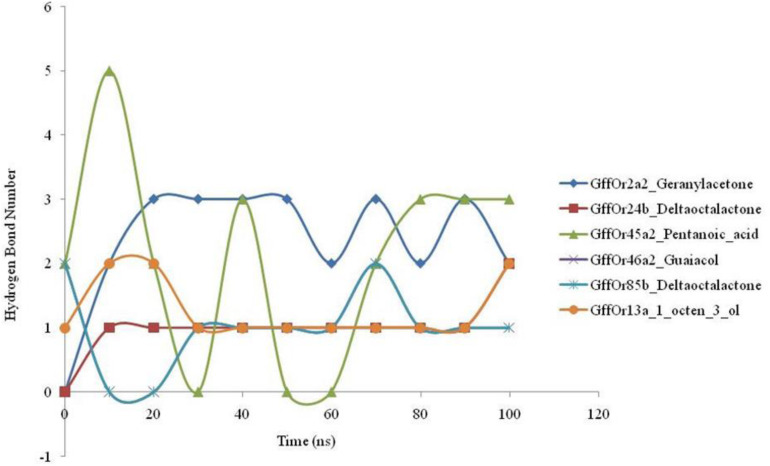
Time-lapse curves highlighting the changes observed in the number of hydrogen bonds between the receptors and the studied ligands during the course of 100-ns MD simulations.

The 100-ns MD simulations were performed for the validation of the docking based generated parameters and the patterns of the hydrogen bonding between the protein and ligand were studied during the course of MD simulations (Figure 4). MD simulations highlighted the changes observed in the structure of the studied protein with the highest structural stability observed in the GffOr85b_δ-octalactone complex. The hydrogen bonding is involved in a diversity of cellular functionalities, as it regulates the molecular interactions in the metabolic processes. Therefore, the understanding of the molecular functions such as ligand binding effects requires the analyses of the hydrogen bond perturbations. The GffOr2a2_Geranylacetone showed the presence of three H-bonds, while in GffOr24b_δ-octalactone one H-bond was observed. In the GffOr45a2_Pentanoic_acid complex, the hydrogen bonds were observed until the 20-ns time period and up to 70 ns a lower number of H-bonds, but afterward, a constant three H-bonds were observed (Figure 4). Moreover, GffOr46a2_Guaiacol, GffOr85b_δ-octalactone, and GffOr13a_1_octen_3_ol showed similar H-bond patterns with the number raised to one.

#### Evaluation of Complex Compactness and Stability

**FIGURE 5:**
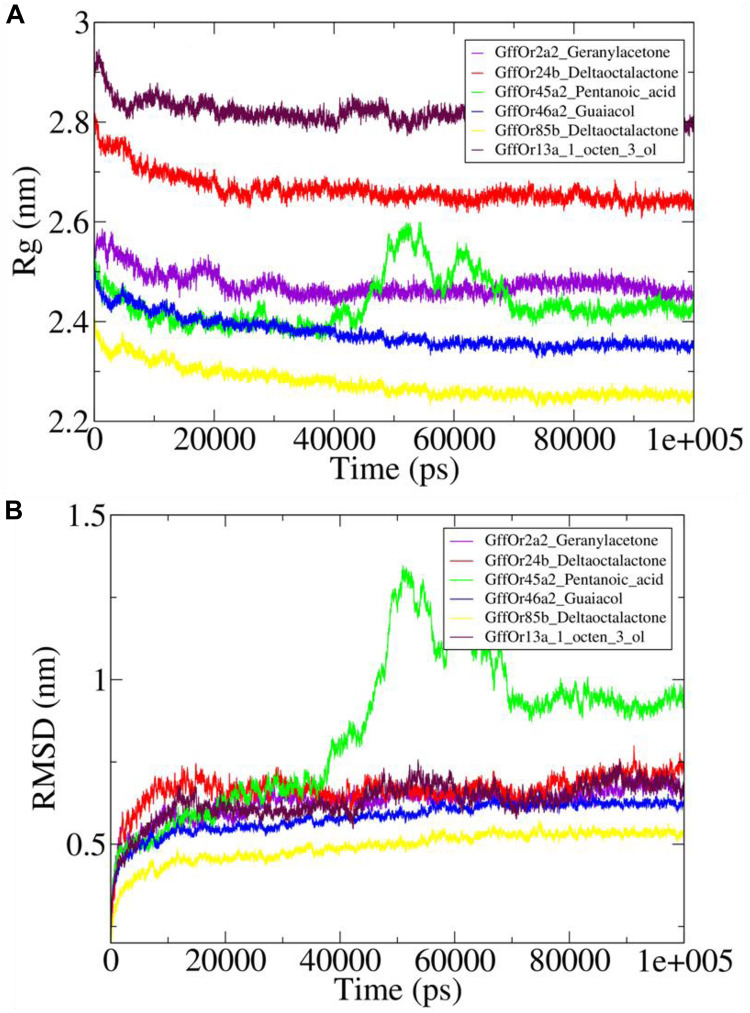
The complexes stability assessment curves with **(A)** highlighting the changes observed in the pattern of the radius of gyration (Rg) and **(B)** illustrating the fluctuations in the patterns of root mean square deviation (RMSD) values.

The Rg was computed using gmx gyrate module of the GROMACS which illustrated the stability of the protein by calculating the compactness of the system, which is the reflection of the stable nature of the protein (Figure 5A). The variations in the Rg values were observed around 2.5 nm for the GffOr2a2_Geranylacetone system, while in GffOr24b_δ-octalactone the Rg values fluctuated between 2.6 and 2.7 nm, which were higher than in the rest of the system, indicative of less compactness in the respective system (Figure 5A). In GffOr45a2_Pentanoic_acid, the Rg values fluctuated around 2.4 nm up to 40 ns but afterward rose up to 2.6 nm and then gradually decreased and became stable between 2.4 and 2.5 nm after the 70-ns time period (Figure 5A). Similarly, for GffOr46a2_Guaiacol, the Rg values varied around 2.4 nm, but in GffOr85b_δ-octalactone, the highest compactness was observed, which was indicative of the obtained Rg values present between 2.2 and 2.3 nm. Moreover, the GffOr13a_1_octen_3_ol system showed the lowest compactness among the studied complexes, indicating a lesser degree of protein folding (Figure 5A).

Furthermore, the conformational stability of the studied docked systems was further assessed using RMSD values (Figure 5B). It was observed that GffOr45a2_Pentanoic_acid interaction was the least stable during 100-ns MD simulations, with RMSD values continuously fluctuating, rising sharply after the 40-ns time period but stabilized after 70 ns (Figure 5B). GffOr2a2_Geranylacetone, GffOr24b_δ-octalactone, and GffOr13a_1_octen_3_ol showed a relatively similar pattern, indicating similar stability was present in the systems, but GffOr46a2_Guaiacol was slightly more stable than the respective systems (Figure 5B). GffOr85b_δ-octalactone achieved the highest stability, as observed from the measured RMSD values (Figure 5B).

#### Time Evolution of System Energies

**FIGURE 6:**
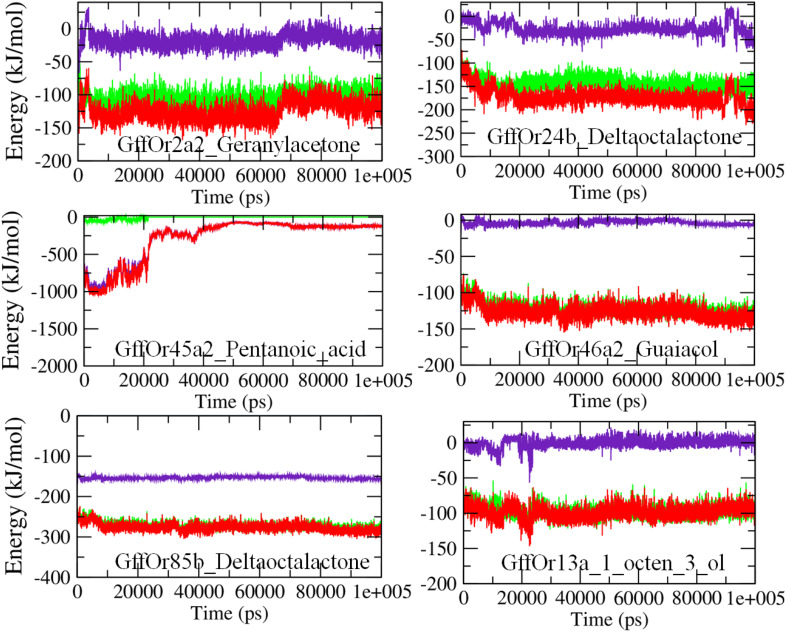
The MM/PBSA based energy curves highlighting the changes observed in the pattern of the interaction energies observed between the studied receptors and ligands during the course of 100-ns MD simulations. Green, van der Waals energy; indigo, electrostatic energy; red, total energy.

The molecular mechanics Poisson–Boltzmann surface area (MM/PBSA) based algorithm was used to calculate the interaction energies, as an indication of the binding strength between the proteins and the ligands (Figure 6). The MM/PBSA calculates the free energies of the interactions by combining three energetic terms: potential energy in the vacuum, solvation energies in the implicit solvation model, and configurational entropy associated with complex formation (Kumari et al., 2014). In the GffOr2a2_Geranylacetone complex, the total free energy of interactions was observed between −100 and −150 kJ/mol, while for GffOr24b_δ-octalactone the energy was observed around −200 kJ/mol. In the GffOr45a2_Pentanoic_acid system, the total energy was observed between −1000 and −500 kJ/mol up to 20 ns but afterward, the interaction became unfavorable, indicating changes in the energy values (Figure 6). Similarly, in GffOr46a2_Guaiacol, the total free energy of binding observed was around −150 kJ/mol. The lowest interaction energies of around −300 kJ/mol were observed in GffOr85b_δ-octalactone system, indicating the relatively favorable nature of binding between the respective protein and ligand. In addition, the total energy of GffOr13a_1_octen_3_ol observed was between −50 and −100 kJ/mol, with a lesser contribution of the electrostatic energy.

### Docking-Based OR–Ligand Interaction Site Studies

**FIGURE 7:**
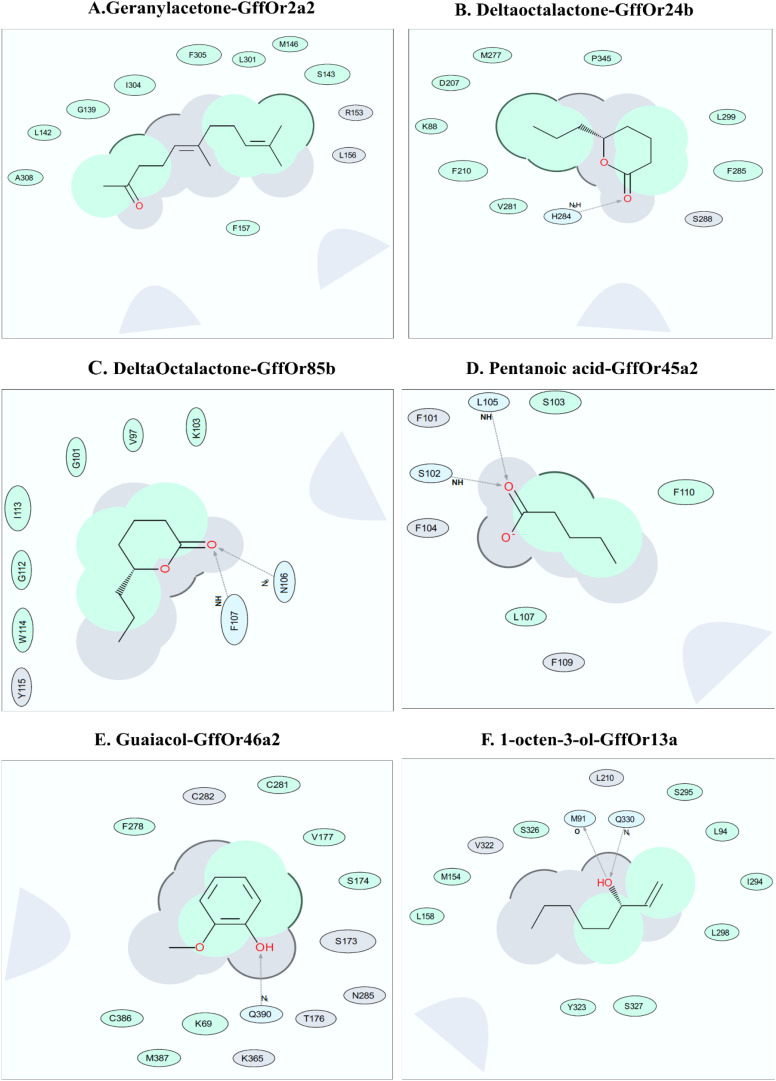
Bioinformatics analysis for the interaction of the different ligands with their putative odorant receptors. Amino acid residues in interaction within 5 Å of ligands as depicted by the ligand interaction diagram for pairs **(A)** Geranyl acetone-GffOr2a2, **(B)** δ-octalactone_GffOr24b, **(C)** δ-octalactone-GffOr85b, **(D)** pentanoic acid-GffOr45a2, **(E)** Guaiacol-GffOr46a2, and **(F)** 1-octen-3-ol-GffOr13a. Generated using ICM-Pro software (Ruben Abagyan, 1994) (version 3.8-7, MolSoft LCC, San Diego, CA, United States, www.molsoft.com). Green shading represents the hydrophobic region; white dashed arrows represent hydrogen bonds; gray parabolas represent accessible surface for large areas. The broken thick line around the ligand shape indicates an accessible surface. The size of the residue ellipse represents the strength of the contact.

To explore the binding interfaces of the selected top five scoring docked complexes, a 2-D interaction diagram was built using ICM-Pro software version 3.8-7 (MolSoft LCC, San Diego, CA, United States^9^). Several predominantly hydrophobic interactions were observed between the tested ligand and putative receptor binding site residues. Our predicted ligand-binding pockets consisted mostly of hydrophobic residues, with a few hydrogen bonds, supporting the MD data, except the Geranylacetone–GffOr2a2, which did not show any hydrogen bond formation, for unknown reasons. We found that geranylacetone could possibly interact with GffOr2a2 at up to nine possible interactions sites, including at G139, S143, I 304, and F305 (Figure 7A). δ-Octalactone–Or24b and δ-octalactone–GffOr85b showed respectively six and eight interaction sites. The residues F210, M277, F295, I299, and P345 could be potential interaction sites. Likewise, the complexes δ-octalactone–Or24b, I113, C101, W114, and G112 are predicted potential interaction sites. Also, two H-bonds were observed in δ-octalactone–GffOr85b, but one H-bond in δ-octalactone–Or24b (Figures 7B,C). The less stable complex, pentanoic acid–GffOr45a2, showed possible interactions with F110, S103, and L107. Additionally, two H-bonds were observed at I105 and S102 for this complex (Figure 7D). The complex Guaiacol–Or46a2 showed five possible interaction residues, K69, S174, V177, C291, and M387, and one H-bond at Q390 (Figure 7E). 1-Octen-3-ol-GffOr13a revealed nine (L94, M154, L158, I294, L298, S295, S326, S327, and Y323) interaction sites. We also noted two H-bonds at residues M91 and Q330 for 1-octen-3-ol-GffOr13a complex (Figure 7F).

### Olfactory Sensory Neuronal Response

**FIGURE 8:**
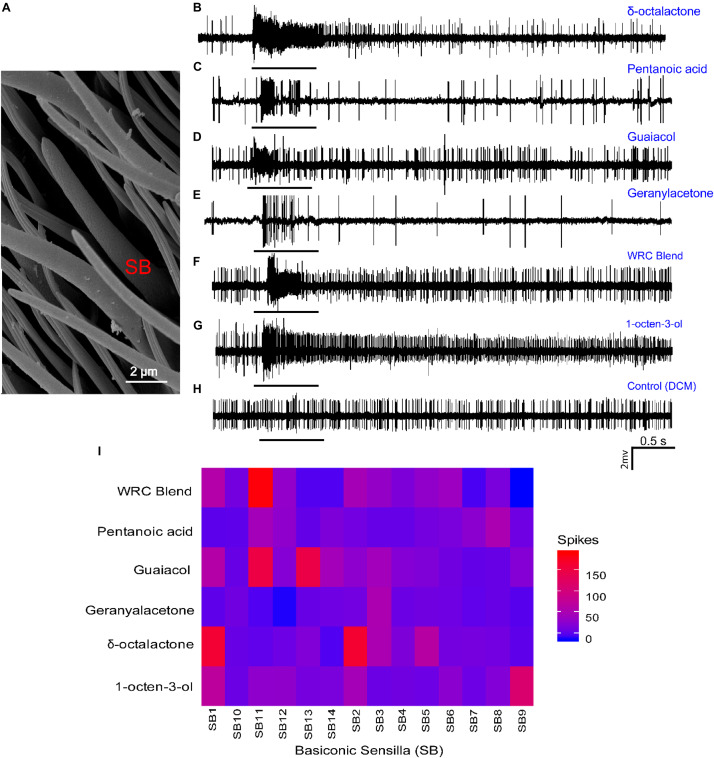
Cellular response patterns of basiconic sensilla of *G*. *f*. *fuscipes* to different chemicals. **(A)** Scanning electron micrograph of the basiconic Sensilla (SB) of *G*. *f*. *fuscipes*. **(B*–*H)** Representative single-sensillum recording (SSR) traces, showing responses to the indicated odorant and the control (DCM). **(I)** Heatmap of OSN response patterns of 14 basiconic sensilla of *G*. *f*. *fuscipes* elicited by different odors used in DREAM techniques, generated using R software version 3.5.1 (R Core Team, 2018), www.R-project.org, edited using Adobe illustrator CS5.1.

Having identified the change in transcript levels of OR mRNA, followed by docking and MD analysis, and identified putative OR, we next analyzed the physiological response of the WRB, the four components, and 1-octen-3-ol using single-sensillum recording techniques. Dilution levels at 10^–3^ (v/v) were used to validate the presence of receptor proteins in the olfactory sensilla of *G*. *f*. *fuscipes*. The electrophysiological recording was performed only for large basiconic sensilla (Figure 8A); we found these sensilla types distributed well all over the antennal region, and basiconic sensilla house ORs responding to host odors (Figure 8A). From the targeted sensilla (*n* = 14), most of the sensilla housed one to two OSNs per sensillum based on their spike amplitude. The targeted sensilla consistently showed spontaneous action potential. The odor–OSN interaction resulted in different response dynamics and spike magnitudes. For example, some odor (1-octen-3-ol) resulted in a prolonged response and activated all tested sensilla, while others resulted in a phasic response, i.e., geranyl acetone (Figure 8E). δ -Octalactone elicited a response of 172 spikes/s, while 1-octen-3-ol elicited a response of 102 spikes/s in some sensillum. Similarly, the WRB blend elicited a response of 188 spikes/s in one sensilla. However, geranyl acetone did not elicit a strong response; the maximum from all tested sensilla was 53 spikes/s. Pentanoic acid similarly elicited a maximum response of 74 spikes/s. Guaiacol, which is a WRB component, resulted in up to 159 spikes/s. The OSNs housed in the targeted sensillum varied in their response spectrum; some were selective even at the tested concentration (SB14, SB7, and SB10), while others responded to most of the tested odors (SB1, SB2, and SB11) (Figure 8I). Furthermore, the WRB blend elicited a response distinct from its constituents’ response (Figures 8F,I).

## Discussion

In this study, we analyzed how tsetse repellent components are coded in the olfactory sensilla of the tsetse fly *G*. *f*. *fuscipes*. The tsetse repellents showed strong antifeedant activity to *G*. *f*. *fuscipes*. Of the WRB components, pentanoic acid, δ-octalactone, and geranylacetone contributed strongly to the antifeeding effect, with geranylacetone appearing to serve as the key odor contributing greatly to the feeding inhibition effect of the WRB. Nonetheless, our results show that these three components are essential and sufficient to elicit the strongest antifeedant activity of the WRB. As removal of guaiacol did not affect the feeding of *G*. *f*. *fuscipes* on treated blood, our results suggest that guaiacol plays no role in the antifeedant effect of the WRB.

Field trap capture studies showed that removal of δ-octalactone from the blend reduced the repellency of the WRB on *G*. *pallidipes* (Bett et al., 2015). Likewise, when tested singly, pentanoic acid and geranyl acetone were found to reduce trap catches of *G*. *pallidipes* (Bett et al., 2015). A recent study on *G*. *f*. *fuscipes* under field conditions showed that WRB reduced trap catches by 33% and the different components had a different contribution to spatial repellency of the WRB (Mbewe et al., 2019). Our results show the additional effect of WRB as an antifeedant, besides on its spatial repellency. Similar results have been found in mosquitoes using DEET, whereby DEET affected the feeding behavior of mosquitoes, in addition to its spatial repellency (Wei et al., 2017). Similarly, geosmin inhibited the feeding of *D*. *melanogaster*, functioning as an antifeedant, operating via the olfactory system, besides its strong repellency (Stensmyr et al., 2012). Furthermore, in mosquitoes, olfactory sensilla are associated with other organs such as the stylet, suggesting that olfaction plays a role in odor perception at close range (Won Jung et al., 2015). Because tsetse flies and other insects express gustatory receptors (GRs) over their entire bodies (Vosshall and Stocker, 2007; Obiero et al., 2014; Macharia et al., 2016), taste might also play a role in blood feeding as well as inhibition, at short range in feeding that needs further investigation.

We characterized the change in transcript expression change of all the ORs in the antenna of *G*. *f*. *fuscipes* to determine which ORs are involved in the detection of WRB. We found that in *G*. *f*. *fuscipes*, following *in vivo* exposure to WRB volatile chemicals in an open cage, OR genes that interacted with the given odor responded in two ways. Some of the OR mRNA transcripts were downregulated, while others overexpressed their mRNA transcripts as reported previously (Weid et al., 2015; Ibarra-Soria et al., 2017; Dewan et al., 2018; Koerte et al., 2018). To account for this significant reduction in mRNA transcripts, we hypothesize that it has an adaptive function in response to olfactory overstimulation. It would represent a form of neural plasticity that would desensitize neurons, similar to other molecular adaptations that take place at the olfactory transduction or processing levels (Leinders-Zufall et al., 2000; Kato and Touhara, 2009; Getahun et al., 2012; Weid et al., 2015). Since a previous dose–response experiment in *D*. *melanogaster* after odor exposure did not show a change in sensitivity (Koerte et al., 2018), we cannot rule out this hypothesis without carrying out ecological setting studies that challenge the OSN sensitivity.

Additionally, mRNA transcripts of some ORs significantly increased by up to a 10-fold change. Similarly, Ibarra-Soria et al. (2017) and Koerte et al. (2018) showed that some OR mRNA transcripts increased due to odor exposure. The differential (up and down) regulation in transcript levels of the various ORs is not clear, but it shows their involvement in the detection of the odor to which the insects were exposed. Similarly, Ibarra-Soria et al. (2017) showed stimulation with odors resulted in modulation of the mRNA transcript levels in many OR genes, whereby they were upregulated in some and downregulated in others. The opposite change in OR mRNA transcript to the same compound shows there is an individualized response in the OSNs of *G*. *f*. *fuscipes*, which might provide the olfactory system freedom of odor coding and neuronal diversity. The current hypothesis about up- and downregulation of OR mRNA transcripts is not clear. According to previous studies (Weid et al., 2015; Koerte et al., 2018), upregulation occurs because of OSN inhibition. Interpreting OR transcript upregulation as occurring because of OSN inhibition is difficult. We showed almost all of the targeted OSN responses were excitatory (Figure 8), which could be due to targeting a subset of sensilla, as demonstrated in the present study. Furthermore, because in the present study we stimulated the entire receptor repertoire (the whole sensilla), we could potentially have generated a mixed response. Our alternative hypothesis is OSN plasticity to handle the high influx of odors encountered. For example, in the moth pheromone system, sensillum housing OSNs that respond to the major pheromone component have a high pore density accompanied by high OR expression to handle the maximal ranges of molecular flux imparted by major pheromone components in every plume strand (Baker et al., 2012). Furthermore, prior exposure to a given odor that creates a rich olfactory experience shapes the OSNs tuned to the exposed odor to be more sensitive and enhances its discriminative power, showing exposure-dependent adaptation at the level of the receptor neuron (Iyengar et al., 2010).

Deorphanization of ORs of non-model insects is a challenge, but the DREAM method developed by Weid et al. (2015) allows for its use in non-model insects, such as *G*. *f*. *fuscipes*. Furthermore, according to Koerte et al. (2018), there is a good correlation between the change in mRNA and receptor–ligand interaction in the model *Drosophila* of about 69%. However, Koerte et al. (2018) also noted the limitations of DREAM in predicting potential receptor false-positive and false-negative results. For example, the change in mRNA is influenced by both concentration and exposure time (Weid et al., 2015); thus the use of a high concentration might produce false-positive results, as ORs are less specific at higher concentrations (Hallem et al., 2004). Additionally, since the change in mRNA is reversible (Weid et al., 2015), if ORs are exposed for a long time the required change in mRNA might not be captured.

In the present study, we combined the DREAM technique with molecular docking and compared the corresponding orthologs of *D*. *melanogaster* to allow us to predict putative ORs. Combining the three methods significantly reduced the number of putative receptors for each odor into a few possible receptors when compared to using the DREAM technique alone (Table 1). Additionally, our molecular docking results showed a strong affinity between ligands and identified putative receptors. The MD results of the top scoring docked OR–ligand complex showed a stable complex and strong binding affinity, which demonstrates the reliability of our docking scores. Hence, the identified putative receptors selected in Table 1 could be some of the receptors of the ligands used in this study. Similarly, the orthologous receptors from *D*. *melanogaster* also responded to the given odors or their analogs of δ-octalactone (DoOR), showing that DREAM combined with molecular docking, followed by ortholog comparison and physiological studies can predict potential receptors for a given odor in a non-model insect such as *G*. *f*. *fuscipes*. Interestingly, *Orco* gene expression was not affected by all the tested odorants, showing it can be used as a reference gene for these ORs. This shows it is not directly involved in these ligand interactions, as previously showed (Nichols et al., 2011), but important for the ORx–*Orco* functionality and behavioral response (Larsson et al., 2004; Getahun et al., 2016; Fandino et al., 2019).

With regard to the correlation of the antifeedant effect with up- and downregulation of mRNA, odors that inhibited blood feeding also affected the mRNA transcript in a mixed response both by up- and downregulation of the mRNA transcript. On the other hand, the WRB components that did not inhibit feeding modified the OR mRNA transcript by upregulating after homology modeling followed by comparison of orthologs. Our results show that odors with strong antifeedant effects and feeding stimulants are coded differently at the molecular level. In the future, it will be important to address how the activation of these receptors elicit repellent and antifeedant behaviors. Various studies in *D*. *melanogaster* showed different mechanisms of repellency, that is, the activation of the dedicated olfactory circuit (Stensmyr et al., 2012). Furthermore, other researchers showed that a given odor valence changed due to its concentration and correlated with the recruitment of additional glomeruli (Semmelhack and Wang, 2009; Strutz et al., 2014) that changed the odor valence from attractant to repellent.

Tsetse flies exhibit a reduced number of ORs as compared to other Dipteran flies (Attardo et al., 2019). We analyzed the WRB, its constituents, and 1-octen-3-ol coding in the large basiconic sensilla expressed on the antenna of *G*. *f*. *fuscipes*. Different response dynamics were elicited by the different tested odors on the same sensillum. The different responses of the given OSN to different odors suggests that physicochemical properties of the constituent odorants may influence their interaction with receptors. Similar to previous findings (Soni et al., 2019), the tsetse attractant 1-octen-3-ol activated most of the tested sensilla, and also resulted in prolonged responses in some sensilla. The WRB blend elicited a distinct response from its individual constituents, showing the integration of olfactory information beginning at the periphery, as also shown in *Drosophila* (Su et al., 2011; Getahun et al., 2012), moths (Kramer, 1992), and in beetles (Nikonov and Leal, 2002). Our results are consistent with other conclusions (Soni et al., 2019) about the absence of a strong response from targeted sensilla.

Our results show that some of the OSNs of *G*. *f*. *fuscipes* are less specific, whereby one OR can respond to multiple ligands, and a single ligand can activate multiple ORs (Figures 2, 8). Similarly, OSNs of *Glossina morsitans morsitans* have been found to be broadly tuned to diverse chemical classes (Soni et al., 2019). Likewise, in other insects, it has been demonstrated that non-pheromone volatiles can activate multiple ORs and non-pheromone receptors can also detect more than one chemical including insect repellents and attractants (Firestein, 2001; Hallem and Carlson, 2006; Bohbot and Dickens, 2012a, b). Recently, in *G*. *morsitans morsitans*, GmmOr9 was shown to respond to chemically diverse odors, acetone, 2-butanone, 2-propanol, and 1-octen-3-ol; the latter activated all targeted sensilla (Chahda et al., 2019; Soni et al., 2019). Similar results were recently found in *G*. *f*. *fuscipes* and *G*. *pallidipes* (Ouedraogo and den Otter, 2018). The odorant concentration (10^–3^, v/v) at which we exposed our flies could have induced responses in the majority of the receptors and might be higher than the ecological concentration they encounter in their natural environment. However, their generalist response has to be challenged by using other physiological setups such as GC-single-sensillum recording (Stensmyr et al., 2012; Dweck et al., 2013) or at low concentration stimulation (Hallem and Carlson, 2006; De Bruyne and Baker, 2008; Silbering et al., 2008; Getahun et al., 2016). However, as previously reported (Soni et al., 2019), the spike number response seems less even when tested with high concentrations as compared to *Drosophila*, for unknown reasons. In the future it will be necessary to characterize their responses using different approaches, such as to express these putative receptors in an empty neuronal system (Chahda et al., 2019). Such a system will enable us to validate the identification of these potential/putative receptors using DREAM combined with molecular docking. Also, it is important to show that the reduction or upregulation in mRNA is associated with a corresponding decrease in OR expression on the dendrites of OSNs in the *G*. *f*. *fuscipes* sensillum shaft, and the opposite in ORs with significantly increased mRNA. The continuous use of only one type of tsetse repellent might also lead to repellent resistance flies, as has been demonstrated in other insects for the well-known repellent DEET (Reeder et al., 2001; Klun et al., 2004; Stanczyk et al., 2010). Thus, the identification of the cellular and molecular targets of this strong spatial repellent and antifeedant, WRB, could lead to the discovery of alternative repellents, by targeting the same receptors.

## Conclusion

In conclusion, our study has demonstrated that WRB has a strong antifeedant effect beside its spatial repellency. Furthermore, the DREAM technique, combined with molecular docking, MD, ortholog comparison, and electrophysiology has enabled us to predict the putative ORs involved in coding of this behaviorally well characterized odorant in the non-model tsetse fly *G*. *f*. *fuscipes*. Our molecular and physiological analysis of OR mRNA alteration patterns evoked by repellent and attractant odorants suggests that they vary at the molecular and cellular level by the identity of the activated ORs.

## Data Availability Statement

All data generated and analyzed during this study are included as figures and as additional files. Raw data can be made available after acceptance.

## Author Contributions

SD designed and conceptualized the experiment, generated data, and analyzed the data. MG conceptualized and designed the experiment, contributed to the data generation, and supervised. MS contributed to the MD analysis. AC, DM, and BT contributed to the experimental design and supervision and reviewed and commented on the manuscript. SD and MG wrote the manuscript. All authors commented on the manuscript.

## Conflict of Interest

The authors declare that the research was conducted in the absence of any commercial or financial relationships that could be construed as a potential conflict of interest.

## Footnotes

1 http://www.syntech.nl

2 http://www.ockenfels-syntech.com

3 https://www.vectorbase.org

4 https://flybase.org

5 https://servicesn.mbi.ucla.edu/SAVES/

6 http://www.molsoft.com/

7 http://bioinf.cs.ucl.ac.uk/psipred/

8 http://bioinf.cs.ucl.ac.uk/psipred/

9 www.molsoft.com

## Supplementary Material

TABLE S1 Primers used for RT-qPCR experiments.

TABLE S2 Protein quality check and 3-D structure validation using ProSA *Z*-score and Ramachandran plot statistics obtained from Procheck.

TABLE S3 Alteration of *G. f. fuscipes* OR mRNA and *D. melanogaster vs G. f. fuscipes* orthologs genes response comparison.
