# Supplementary material for: Cellular and Molecular Targets of Waterbuck Repellent Blend Odors in Antennae of Glossina fuscipes fuscipes Newstead, 1910
Source: Front Cell Neurosci. 2020 Jun 3;14:137. doi: 10.3389/fncel.2020.00137 (PMC7283967; doi:10.3389/fncel.2020.00137)
Supplement: TABLE S1 — Primers used for RT-qPCR experiments. [file Table_1.docx]

Cellular and Molecular targets of Waterbuck Repellent Blend (WRB) odours in antennae of *Glossina fuscipes fuscipes* Newstead, 1910

Supplementary data:

**Table S1**: Primers list for RT-qPCR experiments

| GffOr2a1_F | GCCTACAGCTACCAGAGCTA |
| --- | --- |
| GffOr2a1_R | TACAGGTGGTTCTCGATCTT |
| GffOr24b_F | GCACATCAAGAGGAAGTTCT |
| GffOr24b_R | GTAGATGATGTCCTGCTGGT |
| GffOr2a2_F | TCATCTTCGAGTACCTGGAC |
| GffOr2a2_R | GTAGATGATGTCCTGCTGGT |
| GffOr33b_F | TGGAGATGTTCCCCTGCTAC |
| GffOr33b_R | TCCTGCCTGTAGTTCCTGCT |
| GffOr43a1_F | GCAGTTCGTGTACCTGCTGA |
| GffOr43a1_R | GCTGATCTCGATGTCCCTGT |
| GffOr43a2_F | GTGATCAGGAACCAGGAGGA |
| GffOr43a2_R | GTGTACTTGGGCAGCCTGTT |
| GffOr42b_F | TGATGATCGACTTCCACTGC |
| GffOr42b_R | GGTGCATGATGTTCTTGTGG |
| GffOr45a1_F | CCGACCAGATCTTCCACTTC |
| GffOr45a1_R | ATCAGCAGGCTCTTCCTCAG |
| GffOr45a2_F | GAGGAGCTTCAACCTGATCG |
| GffOr45a2_R | CCGAAGTCGTCCCTGTACTC |
| GffOr45a3_F | TACTGGACCTACGGCCTGAT |
| GffOr45a3_R | CCAGCTTGTTCATCCTGTTG |
| GffOr46a2_F | CTACGAGTACCTGGGCTTCG |
| GffOr46a2_R | TTCAGCTCCTCGTTGATGTG |
| GffOr59a_F | CAAGGACAAGCAGAACCACA |
| GffOr59a_R | TGATGAACTTCAGGCTGGTG |
| GffOr67c1_F | AGCAGAAGAGCTTCGAGGTG |
| GffOr67c1_R | GGTAGGGCAGCTTGTAGTCG |
| GffOr67d1_F | ACACCGTGTGCTTCATCAAC |
| GffOr67d1_R | GATGTTCACCAGGTGCTTCA |
| GffOr67d5_F | ACTTCTGGGTGATCCTGGTG |
| GffOr67d5_R | GGCTGTCGTAGATGATGTCG |
| GffOr67d6_F | ATCGGCTTCCTGTACACCAT |
| GffOr67d6_R | TGCTCGATCAGGATGAACTG |
| GffOr74a_F | CAGGAGGTTCAGGAAGATCG |
| GffOr74a_R | CCAGAAGATGTAGGCGAAGC |
| GffOr7a2_F | AGAACGACGAGAAGCAGAGC |
| GffOr7a2_R | CGTTGCTGAAGAAGCTGATG |
| GffOr82a_F | TGAGCATCTGCTTCACCAAC |
| GffOr82a_R | TCGTTGTCCCTCTTCAGCTT |
| GffOr85b_F | GCGCCTTCATCTTCACCTAC |
| GffOr85b_R | GGCTCTTGAAGTCCTCGTTG |
| GffOr85c_F | ATGAGCTACATCGGCTTCGT |
| GffOr85c_R | TGCCTCACGTAGATGTCCAG |
| GffOr85d_F | GGCCATGCTGAAGATCAAG |
| GffOr85d_R | GATGTTGATGTCGCTGCTGT |
| GffOr85e_F | AGCTTCAAGGACAGCGAGAG |
| GffOr85e_R | GCTGAACAGGATCAGGAAGC |
| GffOr88a_F | CTGACCTGGGACCACATCTT |
| GffOr88a_R | GGTCTCGTTCAGCTTCTTGG |
| GffOr94b_F | CCAGTACATGTGGGTGATGG |
| GffOrco_R | AATAAGCCGCCTCCATAACC |
| GffOrco_F | ACTTGTTGCCGACCTTATGC |
