# Supplementary material for: Cellular and Molecular Targets of Waterbuck Repellent Blend Odors in Antennae of Glossina fuscipes fuscipes Newstead, 1910
Source: Front Cell Neurosci. 2020 Jun 3;14:137. doi: 10.3389/fncel.2020.00137 (PMC7283967; doi:10.3389/fncel.2020.00137)
Supplement: TABLE S2 — Protein quality check and 3-D structure validation using ProSA Z-score and Ramachandran plot statistics obtained from Procheck. [file Table_2.docx]

**Table S2**: Protein quality check and 3D structure validation using ProSA Z-score and Ramachandran plot statistics obtained from Procheck.

| Olfactory Receptors | ProSA  z-score | Percentage of  residues in  most favored  region | Percentage of  residues in  additionally  allowed region | Percentage  of residues  in generally  allowed region | Percentage  of residues  in disallowed  regions | Z-score | G-factor |
| --- | --- | --- | --- | --- | --- | --- | --- |
| DmOr7a | -4.16 | 88.1 | 8.3 | 2.1 | 1.6 | -5.826 | -1.15 |
| DmOr13a | -4.32 | 89.5 | 8.5 | 1.0 | 1.0 | 0.415 | -0.04 |
| DmOr19a | -4.34 | 91.3 | 6.7 | 1.4 | 0.6 | 0.704 | 0.00 |
| DmOr22a | -3.87 | 89.2 | 8.9 | 1.1 | 0.8 | -0.267 | -0.02 |
| DmOr35a | -5.51 | 86.7 | 9.8 | 1.6 | 1.9 | -5.984 | -1.27 |
| DmOr43a | -5.8 | 93.5 | 5.6 | 0.6 | 0.3 | 0.873 | 0.05 |
| DmOr45a | -4.9 | 94.4 | 4.4 | 0.6 | 0.6 | 0.539 | 0.08 |
| DmOr59c | -3.09 | 83.8 | 11.3 | 1.8 | 3.1 | -6.586 | -1.23 |
| DmOr67a | -6.14 | 91.7 | 7.5 | 0.5 | 0.3 | 0.374 | 0.06 |
| DmOr69a | -3.01 | 90.8 | 7.2 | 0.8 | 1.1 | 0.569 | -0.03 |
| DmOr71a | -4.57 | 91.0 | 6.9 | 1.2 | 0.9 | 0.561 | -0.01 |
| DmOr85c | -5.27 | 92.9 | 6.3 | 0.5 | 0.3 | 0.706 | 0.08 |
| DmOr85d | -4.97 | 89.8 | 7.6 | 1.8 | 0.8 | -0.036 | -0.07 |
| GffOr2a2 | -4.28 | 91.8 | 6.5 | 0.5 | 1.2 | 0.519 | 0.02 |
| GffOr7a2 | -3.92 | 89.3 | 7.1 | 2.7 | 0.8 | 0.019 | 0.01 |
| GffOr13a | -3.78 | 89.6 | 8.6 | 1.4 | 0.5 | 0.152 | -0.00 |
| GffOr24b | -3.07 | 87.5 | 9.8 | 1.8 | 0.9 | -0.974 | -0.28 |
| GffOr33b | -3.85 | 91.5 | 7.4 | 0.5 | 0.5 | 0.839 | 0.07 |
| GffOr43a1 | -2.86 | 84.3 | 11.1 | 2.0 | 2.6 | -6.052 | -1.24 |
| GffOr42b | -3.82 | 91.1 | 7.8 | 0.9 | 0.3 | 0.412 | 0.03 |
| GffOr45a2 | -1.95 | 86.8 | 8.3 | 3.4 | 1.4 | -0.690 | -0.1 |
| GffOr45a3 | -4.31 | 79.9 | 14.3 | 2.6 | 3.2 | -5.708 | -1.58 |
| GffOr46a2 | -5.4 | 87.3 | 9.0 | 2.7 | 0.9 | -0.587 | -0.07 |
| GffOr59a | -3.81 | 89.5 | 9.6 | 0.6 | 0.3 | 0.899 | 0.06 |
| GffOr67d1 | -1.37 | 89.4 | 6.1 | 2.0 | 2.5 | 0.156 | -0.06 |
| GffOr67d6 | -1.3 | 84.2 | 11.7 | 3.2 | 1.5 | -1.845 | -0.39 |
| GffOr85b | -3.97 | 94.1 | 4.3 | 1.0 | 0.7 | 0.712 | 0.03 |
| GffOr88a | -4.05 | 88.2 | 9.0 | 2.1 | 0.7 | -0.114 | -0.08 |
